# Staff members’ prioritisation of care in residential aged care facilities: a Q methodology study

**DOI:** 10.1186/s12913-020-05127-3

**Published:** 2020-05-14

**Authors:** Kristiana Ludlow, Kate Churruca, Virginia Mumford, Louise A. Ellis, Jeffrey Braithwaite

**Affiliations:** Australian Institute of Health Innovation, Macquarie University, Level 6, 75 Talavera Road, North Ryde, NSW 2109 Australia

**Keywords:** Aged care, Assisted living facilities, Health workforce, Implicit rationing, Missed care, Nursing homes, Prioritisation, Q methodology, Residential facilities

## Abstract

**Background:**

When healthcare professionals’ workloads are greater than available resources, care activities can be missed, omitted or delayed, potentially leading to adverse patient outcomes. Prioritisation, a precursor to missed care, involves decision-making about the order of care task completion based on perceived importance or urgency. Research on prioritisation and missed care has predominantly focused on acute care settings, which differ from residential aged care facilities in terms of funding, structure, staffing levels, skill mix, and approaches to care. The objective of this study was to investigate how care staff prioritise the care provided to residents living in residential aged care.

**Methods:**

Thirty-one staff members from five Australian residential aged care facilities engaged in a Q sorting activity by ranking 34 cards representing different care activities on a pre-defined grid from ‘Least important’ (− 4) to ‘Most important’ (+ 4). Concurrently, they participated in a think-aloud task, verbalising their decision-making processes. Following sorting, participants completed post-sorting interviews, a demographics questionnaire and semi-structured interviews. Q sort data were analysed using centroid factor analysis and varimax rotation in PQMethod. Factor arrays and data from the think-aloud task, field notes and interviews facilitated interpretation of the resulting factors.

**Results:**

A four-factor solution, representing 22 participants and 62% of study variance, satisfied the selection criteria. The four distinct viewpoints represented by the solution were: 1. Prioritisation of clinical care, 2. Prioritisation of activities of daily living, 3. Humanistic approach to the prioritisation of care, and 4. Holistic approach to the prioritisation of care. Participants’ prioritisation decisions were largely influenced by their occupations and perceived role responsibilities. Across the four viewpoints, residents having choices about their care ranked as a lower priority.

**Conclusions:**

This study has implications for missed care, as it demonstrates how care tasks deemed outside the scope of staff members’ defined roles are often considered a lower priority. Our research also shows that, despite policy regulations mandating person-centred care and the respect of residents’ preferences, staff members in residential aged care facilities tend to prioritise more task-oriented aspects of care over person-centredness.

## Background

Healthcare systems are complex, under-resourced and often pressurised environments. Within these systems, clinical and support staff are responsible for providing care to multiple patients with different health conditions and needs, often simultaneously, while completing a variety of associated administrative and care duties within a specified timeframe. High workloads and competing demands can lead to ‘missed care’ (care that is omitted or delayed) [1] as a result of ‘rationing’. Rationing of care, or ‘implicit rationing’ involves “withholding of or failure to carry out necessary nursing measures for patients due to a lack of nursing resources such as staffing, skill mix or time” [2] (p. 228). There is evidence that missed care and implicit rationing are positively associated with medication errors, nosocomial infections, hospital readmissions, urinary tract infections, pressure ulcers, patient falls with injury, mortality, and critical incidences, and negatively associated with quality of care and patient satisfaction [3–5].

**Fig. 1:**
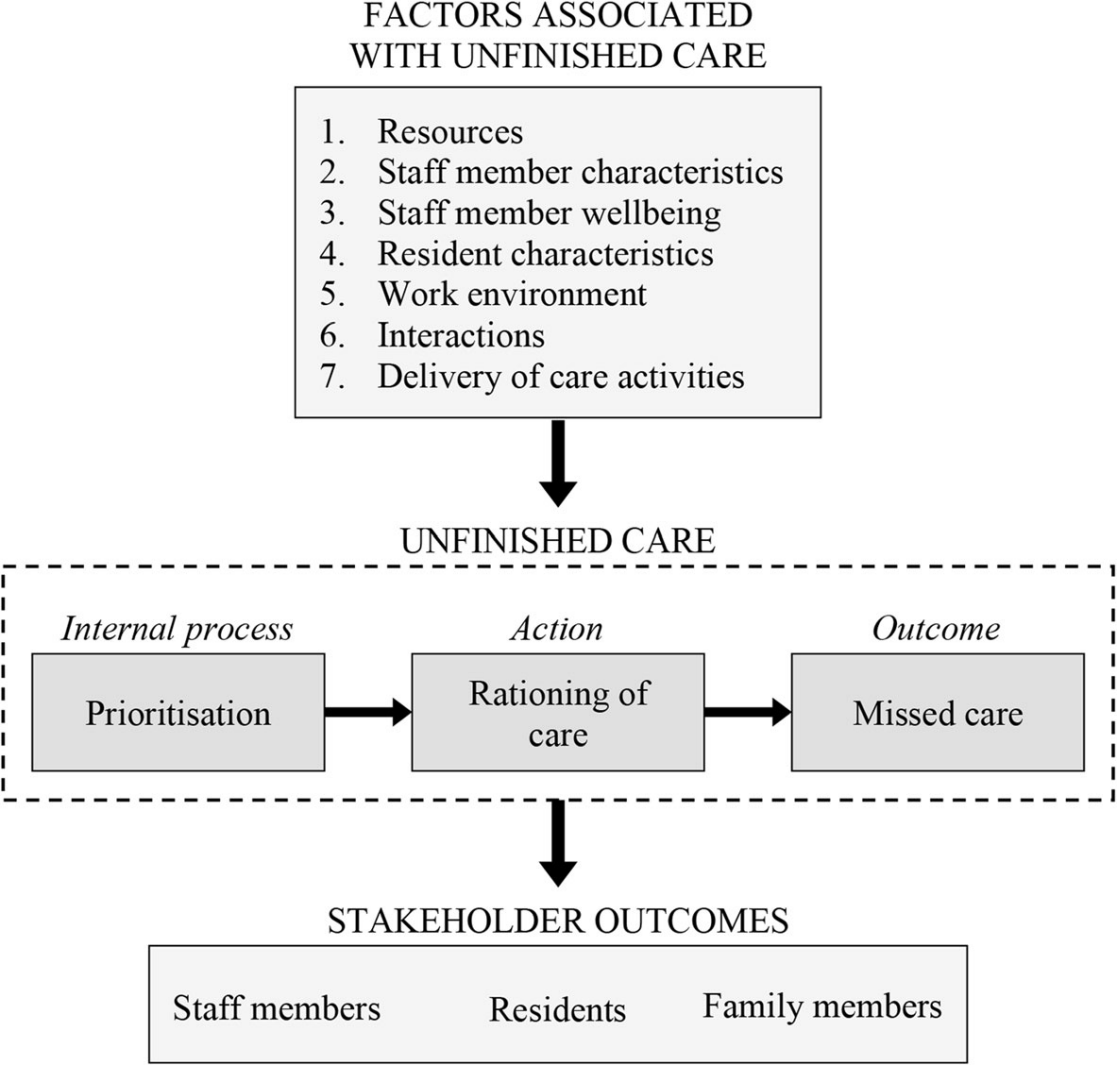
Conceptual model of unfinished care in residential aged care facilities. Authors’ conceptualisation based on [1, 2, 7]

Researchers conceptualise rationed or missed care as a potential consequence of staff members’ ‘prioritisation’ decisions [1, 2]. Prioritisation involves temporally ordering care tasks or problems according to perceived importance or urgency [6]. It is a necessary process that enables staff members to adapt to dynamic and unpredictable situations. In order to manage their workloads, staff members must make judgements about what residents or care tasks should be attended to first and which care activities can be delayed or left undone. As a collective group of concepts, prioritisation, rationing and missed care will be henceforth referred to as ‘unfinished care’ (Fig. 1) [3].

The study of unfinished care originated in acute care settings [8–14], with research predominately conducted in hospitals. More recently, the focus of this research field has expanded to incorporate studies of residential aged care facilities [7]. These facilities are susceptible to unfinished care due to the impact of aging populations on resources [15, 16], staffing issues related to ratios and skill mix [17–19] and a consumer population with complex care needs related to frailty, dementia and multimorbidity [20, 21]. Residential aged care facilities are required to provide social care, pastoral care and meaningful activities in addition to assistance with daily living and clinical care, which raises questions about how these different requirements for providing care to residents are managed in such pressurised environments.

Most research on unfinished care in this setting has focused on implicit rationing (action) or missed care (outcome) [7] (Fig. 1). Previous studies have explored the types of care that are rationed/missed [22], the frequency of rationing/missed care [23, 24], and the factors that influence rationing/missed care [25, 26]. Within this field, very little is known about prioritisation (internal process)*.* A recent integrative review on unfinished care [7] identified only two journal articles [27, 28] that explicitly studied prioritisation of care within residential aged care facilities. Both articles reported on a larger study that interviewed clinical staff members (physicians and nurses) regarding prioritisation dilemmas and prioritisation decisions. The perspectives of non-clinical care staff, who in many cases make up the majority of residential aged care workforces [29], were not included. As prioritisation is an important precursor to missed care and potential adverse patient outcomes, it is important to understand how clinical and non-clinical staff members prioritise the care they provide to residents.

This study formed part of a larger research project exploring prioritisation in residential aged care settings [30]. The objective of the study was to investigate how care staff prioritise the care provided to aged care residents living in residential aged care facilities. The study had two research questions:

1. What are staff members priorities regarding the care they provide to residents?
2. How do staff members prioritise care?

## Methods

### Study design

**Fig. 2:**
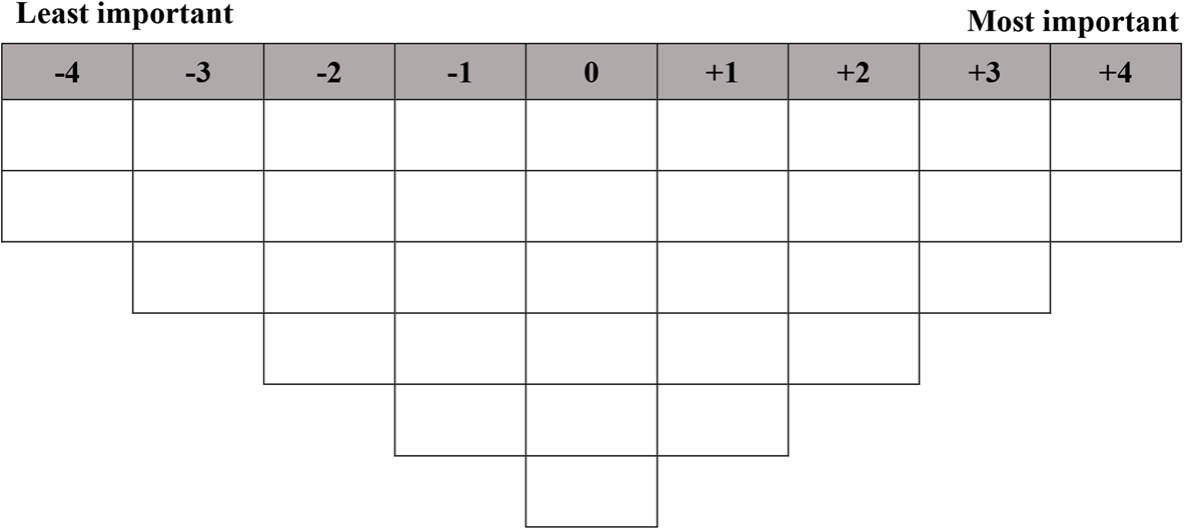
Q sort grid

This was a multi-site Q methodology study of care priorities among staff members working in residential aged care. The study comprised a card sorting activity using Q methodology, a think-aloud task, a demographics questionnaire, and post-sorting and semi-structured interviews. Q methodology is a method used to study subjectivity through the integration of qualitative and quantitative data [31–33]. It involves participants ordering a set of cards (Q sort deck) on a pre-established forced distribution (Q sort grid, Fig. 2), by level of relevance, agreement, or in the case of this study, importance [34]. Participants’ finished Q sorts (the patterns of card placement on the Q sort grid) are then correlated through by-person factor analysis to identify distinct viewpoints, or ‘shared meaning’ (factors) [35, 36]. For a more detailed explanation of Q methodology, we recommend Watts and Stenner’s *Doing Q Methodology: Theory, Method and Interpretation* [37].

Q methodology is an ideal method to address the study objective as it requires participants to decide on the importance of all care activities in relation to each other. Ultimately, it forces participants to prioritise some aspects of care over others. The purpose of the think-aloud task was to provide additional insight into participants’ decision-making processes by asking them to verbalise their thoughts and feelings during the Q sorting activity [38, 39]. The post-sorting interviews [32] focused on individual card placement and enabled the researcher to clarify anything participants said during the think-aloud task. Semi-structured interviews provided information about participants’ personal experiences of having to prioritise care in the past.

### Sample and setting

Study facilities included five Australian residential aged care homes managed by one aged care provider, in New South Wales (NSW) (*n* = 3) and Queensland (QLD) (*n* = 2). Care staff were invited to participate if they were currently employed at one of the five sites, were willing and able to give informed consent, and routinely provided direct care to residents. Purposive sampling, a common convention of Q methodology [40, 41], was used to recruit staff members from different roles across the organisation in order to capture a diverse range of perspectives.

### Materials

The Q sort deck comprised 34 magnetic cards, each representing an aspects of care provided to residents. The cards were developed through a review of the literature [7] and discussions with the management team from one of the participating facilities. Each card comprised a statement (e.g., “Assistance with toileting needs”), a corresponding graphic (e.g., a toilet), and relevant examples (e.g., “Assistance using the toilet” and “Incontinence pads are changed regularly”). The Q sort grid was displayed on a magnetic whiteboard (Fig. 2).

The post-sorting interviews covered three topics: 1. The reasoning behind the placement of salient cards, including cards at the extremes of the Q sort grid, 2. Cards that participants thought were not represented by the Q sort deck, 3. Modifications to the completed Q sort. The demographics questionnaire and semi-structured interview guide were developed for this study and are presented in Additional file 1.

### Procedure

Participants were presented with the Q sort deck, Q sort grid and the following instruction: “Order the cards from what is ‘Least important’ (-4) to you, to what is ‘Most important’ (+4) to you in terms of the care provided to residents.” In order to familiarise participants with the cards and reduce cognitive load, they were first asked to organise the cards into three piles: most/more important, somewhat important, and least/less important. Using the most/more important pile first, followed by the least/less important pile, and then the somewhat important pile, participants organised the cards onto the grid from their highest to lowest priorities.

Participants engaged in the think-aloud task concurrent to the Q sorting activity. After all cards had been placed under the designated ranks on the Q sort grid, participants were asked the post-sorting interview questions and given the opportunity to change the placement of cards before completing the demographic questionnaire and the semi-structured interviews. In order to accurately capture participants’ responses, researcher field notes were composed, study sessions were audio recorded, and photographs of participants’ final Q sorts were taken. Audio recordings were transcribed verbatim.

### Analysis

Data from the Q sorting activity were analysed using established Q techniques, based on inverted factor analysis [42–44]. Unlike traditional factor analysis, where participants’ responses on a number of variables are correlated together (i.e., by-variable), Q factor analysis (i.e., by-person) tests the associations between participants [44]. The purpose of this analysis is to identify ‘factors’ which are clusters of participants who have ordered their cards similarly on the Q sort grid. These factors represent distinct viewpoints on a particular topic, such as prioritisation. PQMethod V.2.35, a purpose-designed statistical program [45], was used to carry out the analysis. Centroid factor analysis was performed to extract factors as it allows for the exploration of all possible factor solutions, as opposed to Principle Component Analysis which delivers the mathematically best solution [35]. The numbers of factors retained in the analysis was determined by the following criteria [35, 46]: greatest amount of variance explained while maximising the number of defining Q sorts (Q sorts significantly loading on a *single* factor [factor loading > 0.45, *p* < 0.01]); factors with eigenvalues greater than 1; and at least two defining Q sorts for each factor. Varimax rotation [36], an automatic rotation process, was then conducted to maximize the study variance explained by the factor solution. For each factor retained in the analysis, PQMethod produced a factor array, which is a representative Q sort based on a weighted average of individual Q sorts loading on a particular factor [36] (see Additional file 2).

### Factor interpretation

While analysis is quantitative, factor interpretation in Q methodology is largely a qualitative process of narrativizing each retained factor into a representative viewpoint. KL consulted with KC and LAE to label and interpret each viewpoint using four information sources: *1. Crib sheets.* Crib sheets [47] summarised the placement of cards at extreme ranks, distinguishing statements and consensus statements. Distinguishing statements refer to cards that have been ranked significantly different in one viewpoint compared to all other viewpoints. Consensus statements are cards that do not significantly distinguish between any two factors*; 2. Participant transcripts.* For each factor, transcripts of the participants who loaded significantly on that viewpoint were examined using NVivo V.12 [48] to situate factor arrays in context; *3. Researcher field notes.* Observations were recorded during study sessions; *4. Colour-coded categorisation system.* The factor arrays were transformed into digital replications of the Q sort grid in order to visually represent the entire viewpoint for each factor. KL devised a colour-coded system to classify cards by care category: clinical care, activities of daily living, respect, psychosocial care, and independence and choice (see Additional files 3, 4, 5 and 6). Inspection of the colour-coded factor array representations illustrated how different types of care were differentially prioritised between participants loading on the different viewpoints.

## Results

Thirty-one staff members participated in the Q sorting activity. Four factors, accounting for 62% of study variance, satisfied the inclusion criteria and were interpreted as narrative accounts of viewpoints. This four-factor solution was defined by twenty-two participants (71%) whose Q sorts significantly loaded on (i.e., correlated with) a single factor (*p* < 0.01). The other nine Q sorts either significantly loaded on more than one factor (*n* = 8) or did not significantly load on any factor (*n* = 1). Demographic information for the total sample and for each factor is presented in Table 1.

**Table 1:** Participant demographics

	Overall (*n* = 31)	Factor 1 (*n* = 10)	Factor 2 (*n* = 4)	Factor 3 (*n* = 3)	Factor 4 (*n* = 5)
**Age range**					
18–25	1 (3.2%)	0 (0%)	1 (25%)	0 (0%)	0 (0%)
26–35	12 (38.7%)	6 (60%)	1 (25%)	0 (0%)	1 (20%)
36–45	7 (22.6%)	1 (10%)	1 (25%)	0 (0%)	1 (20%)
46–55	3 (9.7%)	1 (10%)	0 (0%)	0 (0%)	1 (20%)
56+	6 (19.4%)	1 (10%)	0 (0%)	3 (100%)	2 (40%)
Not disclosed	2 (6.5%)	1 (10%)	1 (25%)	0 (0%)	0 (0%)
**Sex**					
Male	13 (41.9%)	4 (40%)	0 (0%)	2 (66.7%)	1 (20%)
Female	18 (58.1%)	6 (60%)	4 (100%)	1 (33.3%)	4 (80%)
**Australian state**					
New South Wales	17 (54.8%)	8 (80%)	2 (50%)	0 (0%)	1 (20%)
Queensland	14 (45.2%)	2 (20%)	2 (50%)	3 (100%)	4 (80%)
**Primary job position**					
Care Assistant	15 (48.4%)	4 (40%)	4 (100%)	1 (33.3%)	1 (20%)
Registered Nurse	7 (22.6%)	3 (30%)	0 (0%)	0 (0%)	0 (0%)
Lifestyle and Activities Officer	5 (16.1%)	1 (10%)	0 (0%)	0 (0%)	4 (80%)
Pastoral Carer	2 (6.5%)	0 (0%)	0 (0%)	2 (66.7%)	0 (0%)
Facility or Care Manager	2 (6.5%)	2 (20%)	0 (0%)	0 (0%)	0 (0%)
**Length of employment at current facility**					
< 2 years	13 (41.9%)	6 (60%)	0 (0%)	1 (33.3%)	2 (40%)
2–3 years, 11 months	8 (25.8%)	2 (20%)	2 (50%)	1 (33.3%)	1 (20%)
4–5 years, 11 months	4 (12.9%)	0 (0%)	1 (25%)	0 (0%)	2 (40%)
> 6 years	4 (12.9%)	1 (10%)	0 (0%)	1 (33.3%)	0 (0%)
Not disclosed	2 (6.5%)	1 (10%)	1 (25%)	0 (0%)	0 (0%)

Note: Value for factors 1–4 calculated as a percentage of n for each factor

The analysis revealed some correlation between the four factors (Table 2). After reviewing the factor arrays (Additional file 2) and colour-coded care categories (Additional files 3, 4, 5 and 6), analysing participant transcripts, and exploring alternative factor solutions, the research team concluded that retaining all four factors was the most appropriate solution as each factor represented a distinct viewpoint. These viewpoints were named: Viewpoint 1: Prioritisation of clinical care; Viewpoint 2: Prioritisation of activities of daily living; Viewpoint 3: Humanistic approach to the prioritisation care; and Viewpoint 4: Holistic approach to the prioritisation of care.

**Table 2:** Correlation matrix

	Factor 1	Factor 2	Factor 3	Factor 4
**Factor 1**	1.0000	0.3361	0.5493*	0.7008*
**Factor 2**	0.3361	1.0000	0.1075	0.3781
**Factor 3**	0.5493*	0.1075	1.0000	0.5810*
**Factor 4**	0.7008*	0.3781	0.5810*	1.0000

* Two factors are significantly correlated *p* < 0.01

The following section details narratives for each viewpoint. Card names are presented as single quotations, followed by the corresponding rank number on the Q sort grid in brackets, based on the factor arrays. Distinguishing statements at *p* < 0.05 and *p* < 0.01 are indicated with a single and double asterisk, respectively.

### Factor interpretation

#### Viewpoint 1: prioritisation of clinical care: ensuring residents’ health and safety

Viewpoint 1 accounted for 23% of study variance and comprised 10 Q sorts from four Care Assistants, three Registered Nurses, one Activities and Lifestyle Officer, and two Managers. Participants who loaded significantly on this viewpoint prioritised clinical aspects of care, as reflected in the cards ranked as most important: ‘Monitoring/Safety’ (+ 4), ‘Medication management’ (+ 4), ‘Medical condition management’ (+ 3), ‘Staff knowledge’ (+ 3**), and ‘Resident information’ (+ 3**). The prioritisation of clinical care is reflected in the following quote from Participant 2 (Manager):

“*At the end of the day, it’s about what is clinically sound … it is about what is our top priority, which is keeping the residents safe from injury or medical harm and that our staff are knowledgeable about their residents’ medical care need*s.”

All of the Registered Nurses and Managers represented by the four factor solution mapped to Viewpoint 1. These participants explained that clinical care was at the forefront of their care duties. The other five participants loading on this viewpoint acknowledged the importance of providing clinical care, despite it not directly relating to their job responsibilities. Participants indicated that residents were in aged care facilities because they needed help managing medical needs. They reasoned that residents, and older populations in general, often have comorbidities, complex medical problems, cognitive impairment, and depression and anxiety.

Participants spoke about how aspects of care were interrelated and how not attending to certain care tasks could have adverse flow-on effects. For example, Participant 6 (Activities and Lifestyle Officer) explained how problems can escalate:

*“If someone’s constipated, they don't want to eat, they will vomit, and it’s painful, and then they have to pass a hard stool, they get a skin tear in their rectum or worse still they have a rupture, then where are we at? We’ve got a complex medication condition from not toileting.”*

Viewpoint 1 was also characterised by a prioritisation of shared knowledge (‘Staff knowledge’, + 3** and ‘Resident information’, + 3**). Participants reported that it was important for staff members to know about residents’ medical conditions and specific needs (e.g., mobility) in order to provide good care and prevent harm. Participants expressed that residents had a right to know about their medical care—it was their care, their bodies, and they knew best how they felt. Although ‘Family information’ (+ 1) was ordered relatively high, it was ranked lower than the other two knowledge-related cards, as participants said that residents were their priority and their care came first.

Participants tended to base their prioritisation of clinical care on two key issues: safety and the prevention of harm; and ensuring physical and mental health. Aspects of care across the spectrum of importance, from the lowest to the highest placed cards, were linked with these two issues. For example, ‘Medication management’ (+ 4) was important in minimising pain and managing depression and anxiety, ‘Repositioning’ (0), although ranked as a lower priority, was linked to pressure sore reduction and infection avoidance, and ‘Nail care’ (− 3) was described by participants as having limited impact on residents’ health or safety.

Although participants acknowledged the importance of providing residents with independence, all independence-related items (except those part of medical care, i.e., resident decision-making and resident information), were ordered as low priorities. Participants provided three reasons for these decisions. First, choice was not viewed to be as important as medical care. Second, many residents have dementia and as such, experience confusion and an inability to make appropriate choices. Third, affording residents choice and independence could put them at risk, conflicting with participants’ priority of safety.

Concerns over residents’ safety were reflected in participants’ responses to the two lowest ranked cards, ‘Seating choice’ (− 4) and ‘Choice about room environment’ (− 4). In offering choice of seating during group activities, participants worried about safety considerations surrounding mobility aids and wheelchairs, risk of falls and toileting needs. Residents’ choices about their room brought up issues of access and space, safety hazards, and dangers of old furniture. Participants spoke about providing residents with choice and independence “within reason” (Participant 6, Activities and Lifestyle Officer).

A common view held by participants loading on Viewpoint 1 was that although ‘Conversations’ (− 2) with residents was very important, staff members did not have enough time to talk to residents, making it a low priority. For example, Participant 27 (Registered Nurse) said:

*“Because there are other things to get done, we don’t have time, you know? We’d love to sit and chat with them [residents] and sometimes that’s what they need, but we have things to do … other priorities.”*

#### Viewpoint 2: prioritisation of activities of daily living: fulfilling role responsibilities

Viewpoint 2 accounted for 13% of study variance and comprised four Q sorts from Care Assistants. This viewpoint represented participants who prioritised residents’ daily needs, for example ‘Oral care’ (+ 4**), ‘Assistance with meals’ (+ 4*), ‘Bathing and Showering’ (+ 3*) and ‘Personal grooming’ (+ 2**). Attending to daily needs was regarded by participants as vital to preventing medical complications, for example, prioritising ‘Toileting’ (+ 3) in order to avoid urinary tract infections. Due to the personal nature of daily care tasks such as toileting or bathing and showering, participants held that ‘Privacy’ (+ 3) was a priority for resident care.

Participants who mapped to this viewpoint were role-oriented, speaking about priorities in terms of their job responsibilities. They explained that they were in direct contact with residents, providing assistance for those with limited physical abilities, and monitoring residents (‘Monitoring/Safety’, + 2). Participants spoke about being the first ones to notice problems and described examples of their role as information brokers—communicating important information to family, management, Registered Nurses and other staff members, as depicted by the following quote from Participant 7 (Care Assistant):

*“This one [card] is also important, that we have to keep an eye [on], because as we are the ones that are giving shower, and taking care, giving them wash, and applying cream on them, so this one is on us, and if there is any skin damage, or skin tear, any bruises, we are the ones who see first and notify to our RN.”*

This focus on role responsibilities influenced participants’ lower priorities, in particular ‘Medical condition management’ (− 3**), ‘Family information’ (− 3**) and ‘Resident information’ (− 4**). Participants said that they associated clinical care and sharing of medical information with the Registered Nurse’s role, as demonstrated by the following response from Participant 20 (Care Assistant):

*“We don’t have anything to do with the medical side, so it’s left to the RN. We just tell them [residents] to speak to the RN.”*

Despite ranking the majority of clinical care cards as less important, ‘Nutrition’ (+ 2), ‘Monitoring/Safety’ (+ 2) and ‘Medication management’ (+ 2) were seen as important aspects of care as they were connected to participants’ duties (e.g., assistance with meals) or had an impact on the way they delivered care. For example, Participant 20 (Care Assistant) explained that if medication was not provided at the right time, this affected residents’ functioning and mood.

Aspects of care categorised as psychosocial care, for example, ‘Social activities’ (− 2) and ‘Spiritual activities’ (− 3) were also a low priority as participants explained that this was part of other staff members’ roles. The only psychosocial card ranked towards the most important end of the Q sort grid was ‘Emotional support’ (+ 1*). Participants explained that as direct carers, they often encountered residents who were upset, lonely or in a bad mood, and as such, providing emotional support was important to them. Similar to Viewpoint 1, ‘Conversations’ (− 2) was ranked as less important. Participants expressed that there was not enough time to talk to residents as they were busy prioritising their assigned tasks. Participant 17 (Care Assistant) explained:

*“We would like to talk to them [residents] but we don’t have enough time … just taking care of their personal needs, we’re so busy … with showering them, with getting them fed and everything so we don’t really have time to … talk with people.”*

Participants explained that affording residents choices was important, however choice-related cards (‘Meal choice’, 0*; ‘Clothing choice’, − 1*; ‘Seating choice’ −2; ‘Choice about room environment’, −4) were ordered as lower priorities due to the restrictions of certain residents’ needs. For example, Participant 7 explained that ‘Choice about room environment’ (− 4) was a lower priority depending on whether participants needed lifters in their room and how residents’ choices about their room environment impacted available space.

#### Viewpoint 3: a humanistic approach to the prioritisation of care: enhancing residents’ wellbeing in their final years

Viewpoint 3 accounted for 14% of study variance and comprised three Q sorts. Both Pastoral Carers loaded on this viewpoint, as well as one Care Assistant. This viewpoint represented participants who took a humanistic approach to care, prioritising residents’ overall wellbeing, as indicated by some of the higher ranked cards: ‘Emotional support’ (+ 4*), ‘Respect’ (+ 4), ‘Spiritual activities’ (+ 3**), ‘Privacy’ (+ 3); ‘Conversations’ (+ 2), and ‘Independence’ (+ 2). Participants’ humanistic approach to prioritising care was reflected in the language they used throughout the study session. Examples include, “openness to learning”, “what’s worth celebrating”, “meaning in life”, and “sense of their life story”. Residential aged care facilities were described as “the last home” (Participant 22, Care Assistant) or “the last stop” (Participant 19, Pastoral Carer), however, participants emphasised that residents’ “end stage of life” (Participant 19, Pastoral Carer) did not need to be a negative experience, but rather could be filled with human connections, meaningful activities and purpose. Participant 31 (Pastoral Carer) spoke about the importance of promoting a meaningful life for residents:

*“There’s still meaning in life, there’s still activities that they can participate in. They can still have an openness to learning new things, they do—they go to art class, they go to discussion groups, they’re on fundraising committees. So that life isn’t over, that they’re not on the scrap heap. I think just the respect that they get … it’s not over until it’s over and that they can still have a life here.”*

Participants discussed the need to help residents celebrate their lives and add meaning to their time in the care facilities. They also spoke about acknowledging residents’ interests and their life histories—the person they were before they entered residential care. In acknowledging residents as individuals, participants viewed ‘Respect’ (+ 4), ‘Privacy’ (+ 3) and affording residents’ dignity as important parts of the care experience.

Similar to Viewpoints 1 and 2, participants expressed that time constraints were a barrier to engaging in meaningful interactions with residents. Regardless, participants loading on Viewpoint 3 still prioritised ‘Conversations’ (+ 2). Participant 22 (Care Assistant) explained that they would “find time” to chat with residents. Related to interactions with residents was participants prioritisation of ‘Emotional support’ (+ 4*). Participants described taking on a comforting role, particularly for residents who did not have visiting family members. Even small gestures could support residents, for example Participant 22 (Care Assistant) recounted:

*“We are the people that see them [residents] the most, we see them more than the family … sorry I always get emotional. There’s one lady in the morning … she got up in the morning and I said, ‘how are you this morning?’ and she said ‘oh, not feeling well’ and I said, ‘well what do you need?’ and she said, ‘I could do with a hug’. So I said, ‘I’ll get up and give you a hug’. So I gave her a hug and we stood there for a minute or two, and you know, it’s those little things, where you can help somebody and make their day better I suppose.”*

Participants indicated that ‘Emotional support’ (+4*) was especially important for residents in their initial months living in a residential care home, which was a time of adjustment and loss (of family, independence and health). This transition was also viewed as affecting family members. One Pastoral Carer (Participant 31) explained that families often experienced guilt, conflict, and worry when residents first moved into a residential care facility, and that it was important for the family to remain involved in care, to “share memorable times” and participate in activities with residents. This may account for why ‘Attitudes towards family’ (+ 1*) was a higher priority for participants loading on Viewpoint 3.

One of the two highest priorities for this viewpoint was ‘Spirituality’ (+ 4**). Religious beliefs were seen as an important aspect of care for a lot of residents who belonged to a generation that placed high value on religion. Spirituality was also conceptualised as a broader concept, including spiritual connection with “nature, music or art” (Participant 31, Pastoral Carer). Participants reported that different types of care were interrelated and in order to keep residents physically and mentally well, they needed to be spiritually and emotionally looked after. This is illustrated by the following quote from Participant 19 (Pastoral Carer):

*“Spiritual health, mental health and physical health are so, so, so related, so that when your physical health or mental health breaks down, you’re spiritual wellbeing becomes a boost and a support to get you back on track physically and mentally as well.”*

Maintaining residents’ physical health and the provision of clinical care was important to participants, with cards such as ‘Medical condition management’ (+ 3), ‘Monitoring/Safety’ (+ 2) and ‘Medication management’ (+ 1) occupying high ranks. When participants spoke about clinical care, they often related it to residents’ comfort and the importance of minimising pain. Assistance with activities of daily living were a lower priority compared to clinical care, with cards ranked between 0 and − 3. The four choice cards, ‘Seating choice’ (− 4), ‘Clothing choice’ (− 4), ‘Meal choice’ (− 2) and ‘Choice about room environment’ (− 2) occupied some of the lowest ranks on the Q sort grid. Participants held that although the broader concept of ‘Independence’ (+ 2) was a priority, choice cards did not have the same “weight” or “necessity” (Participant 31, Pastoral Carer) as other cards. Choice was “ideal and nice to have” but “not a deal breaker” (Participant 19, Pastoral Carer).

#### Viewpoint 4: a holistic approach to the prioritisation of care: consideration of the whole care experience

**Fig. 3:**
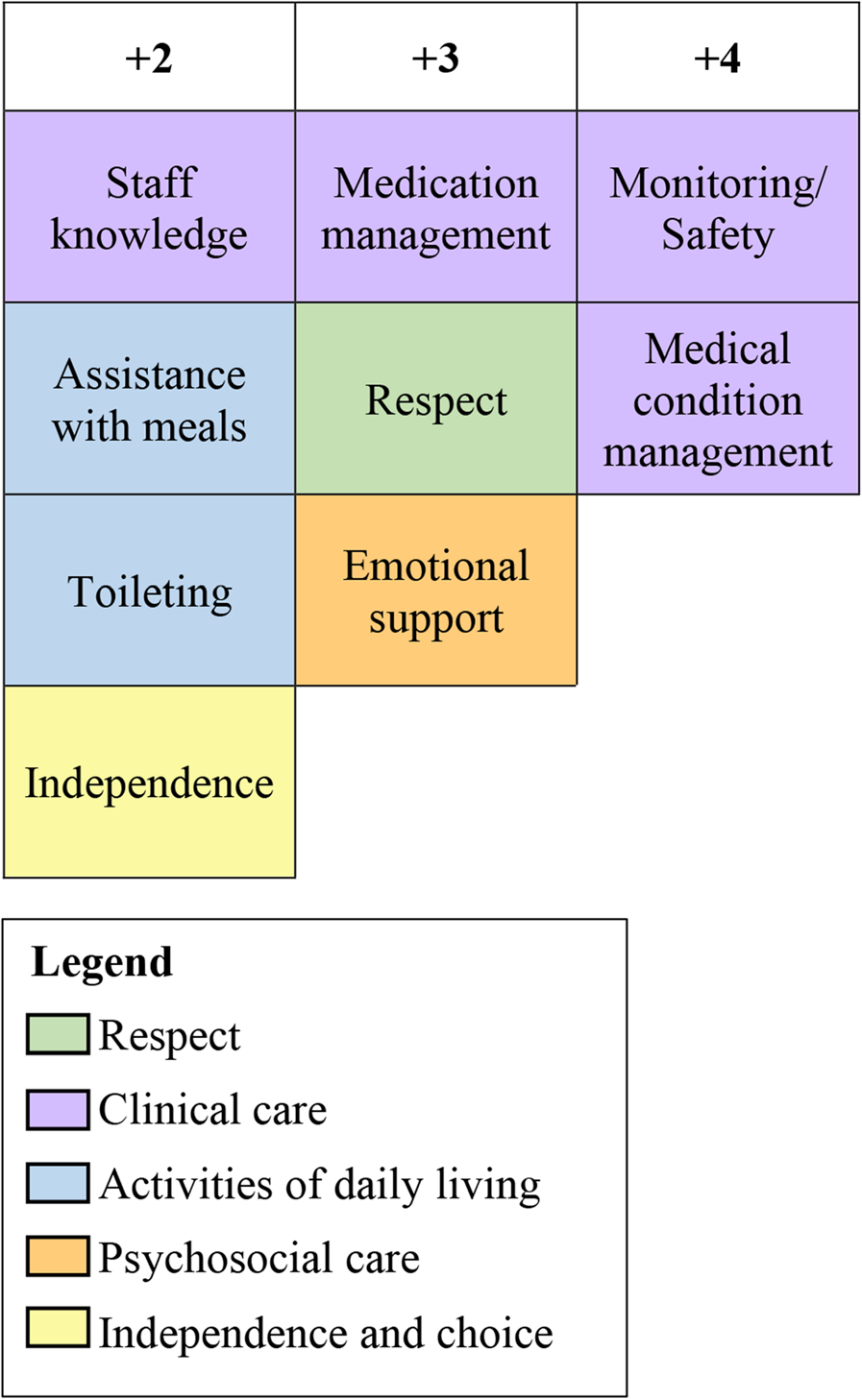
Highest ranked cards in Viewpoint 4

Viewpoint 4 accounted for 12% of study variance and comprised five Q sorts from four Activities and Lifestyle Officers and one Care Assistant. Viewpoint 4 was a composite of Factors 1–3 representing a holistic approach to the prioritisation of care. It was the only viewpoint to have at least one card from each of the five care categories in the highest three ranks (Fig. 3).

Participants loading on Viewpoint 4 shared some of the principles expressed by other participants in the sample. For example, they acknowledged that emotional support was an important part of residents’ care, especially during their transition into a residential aged care facility. They also placed importance on clinical care due to residents’ comorbidities, risk of fall and medication needs. Participants considered different aspects of care to be interrelated, having the potential to impact one another. Those loading on Viewpoints 1–3 ordered the Q cards based on their prioritisation of a specific facet of care (Clinical care, Activities of daily living, and Humanistic aspects of care, respectively), whereas Viewpoint 4 participants prioritised a range of care elements, taking the whole care experience into consideration. Cards that covered broader concepts (e.g., ‘Medical condition management’, + 4; Independence’, + 2; ‘Respect’, + 3) were ranked as top priorities, with more task-focused aspects of care ranked lower. This prioritisation reflected a broad philosophy of holistic care rather than a specific practical approach. One explanation for this pattern of sorting is that the majority of participants loading on this viewpoint were Lifestyle and Activities Officers who were not directly involved in some of the task-focused aspects of care such as ‘Nail care’ (− 4) or ‘Skin care’ (− 2). Participants’ occupation also provided an explanation for why ‘Social Activities’ (0**) was ranked higher in Viewpoint 4 than in other viewpoints. The importance of social care was illustrated by the following quote from Participant 5 (Activities and Lifestyle Officer):

*“So the fact that you have a program that, you know, it’s not childish, it’s a big influence in aging in place. And it’s the things that people have done for many years growing old, like bridge, reading newspapers, watching their favourite programs, classic movies, and the fact that they can see classic movies on a big screen with subtitles in a cinema-like experience once or twice a week, opera, things that they really appreciate.”*

‘Social Activities’ (0**) was not one of the highest ranked priorities, occupying the centre of the Q sort grid. Participants explained that there was no danger in not providing social activities. This was a common justification participants gave for ranking cards lower on the Q sort grid, for example, ‘Nail care’ (− 4) and ‘Privacy’ (− 1**). Whether these care needs were met or not was not considered a “life or death” situation (Participant 5, Activities and Lifestyle Officer). Related to this view was the opinion that some aspects of care could be delayed in favour of attending to more important aspects of care, as Participant 29 (Activities and Lifestyle Officer) explained:

*“These things [higher ranked cards] are about physical, emotional wellbeing, and their self-worth, where the things over here [lower ranked cards] are some things you can fix, come back later on and fix and make it better.”*

The four choice statements, ‘Seating choice’ (− 3), ‘Choice about room environment choice’ (− 3), ‘Clothing choice’ (− 2), and ‘Meal choice’ (− 2) were some of the lowest ranked cards. Participants considered the issue of cognitive impairment when sorting choice-related cards, as shown by the following quote from Participant 26 (Activities and Lifestyle Officer):

*“Residents have choice about their meals, that’s a tough one. I’d probably put that down here, you know with dementia and things, they don’t necessarily make choices that would benefit them.”*

One of the two lowest ranked cards was ‘Attitudes towards family’ (− 4*). Participants acknowledged that being welcoming to family was part of their job, but it was just not a priority. The following response from Participant 26 (Activities and Lifestyle Officer) demonstrates this view:

*“Family members. I mean, it’s important but I wouldn’t say it’s a priority.”*

### Consensus statements

Consensus statements at *p* > 0.01, i.e., cards that did not significantly distinguish between any two factors, included ‘Bowel care’, ‘Choice about room environment’, ‘Repositioning’, ‘Assistance with walking’, ‘Resident decision-making’ and ‘Respect’. The latter three cards were also consensus statements at *p* > 0.05.

### Additional aspects of care

During post-sorting interviews, participants were asked if there were any aspects of care they thought were not adequately represented in the Q sort deck. Eleven participants suggested additional cards, presented in Table 3.

**Table 3:** Additional aspects of care suggested by participants

●Residents’ dignity	
●Residents’ preferred timing of care	
●Pain management	
●Residents’ comfort and having the right equipment for repositioning	
●Cultural diversity	
●Social outings	
●Residents’ experience of transitioning from home to a facility	
●Confidentiality of residents’ personal information and information shared in conversations with residents	
●Involving family in care planning	
●Staff safety/safe working environment	
●The communication of residents’ feedback to staff members	

## Discussion

### Summary of findings

This study investigated what aspects of care staff working in residential aged care facilities prioritise, and how they prioritise care. Four distinct viewpoints were identified: Prioritisation of clinical care, Prioritisation of activities of daily living, Humanistic approach to the prioritisation of care, and Holistic approach to the prioritisation of care. Prioritisation of care was largely influenced by participants’ occupation. Viewpoint 1 represented staff members from a variety of positions, with all Registered Nurses and Managers represented by the factor solution mapping to this viewpoint. Viewpoint 2 comprised only Care Assistants, Viewpoint 3 represented the views of Pastoral Carers in addition to one Care Assistant, and Viewpoint 4 encompassed four of the five Activity and Lifestyle Officers as well as one Care Assistant.

Across the sample, participants reported a deep care for residents and their quality of life. This was reflected in the prioritisation of the ‘Respect’ card, which was positioned in the top four rankings across the four factor arrays. Participants held that it was also important to them that residents were offered choices about their care where appropriate, however other aspects of care often had to be prioritised. Regardless of viewpoint or occupation, participants across the sample consistently ranked residents’ choices, in terms of their room, food, seating and clothes, as lower priorities. Another barrier to providing more person-centred care was a lack of time. Participants explained that having meaningful conversations with residents was important to them but there was not always enough time to prioritise this.

### Role division

Syed and colleagues found broadly similar role orientations to those identified by our research in their ethnographic study on work hierarchies (unequal social relations in the workplace), task orientation (highly focused work that prioritised the completion of tasks), and strict divisions of labour (tasks allocated based on job position, qualifications and skills) in long-term care facilities in Canada [49]. The research team observed that nurses conducted medication administration; support staff (e.g., recreation therapists) were engaged with socialisation and recreational activities; and personal support workers were involved with direct care (e.g., showering and toileting). Divisions of labour and high workloads led to the prioritisation of care duties based on role responsibility. The authors suggested that work hierarchies could potentially impede task sharing by enforcing boundaries between roles.

Daly and Szebehely argued that such a division of labour in residential care homes is partially a consequence of regulations adopted by some governments, which stipulate which occupations can carry out certain care tasks [50]. Their research on the work lives of assistant nurses (licensed or registered nurses) and care aides (e.g., personal support workers, nursing aides) in Sweden and Canada, found differences in the way care was delivered between the two countries. In Sweden, care was more relational and integrated, with care staff carrying out tasks across the spectrum of care (clinical care, personal care, social care, cleaning, cooking), regardless of occupation. In Canada, care was more task-oriented, regulated and formal. Similar to our findings about care prioritisation by role, there were boundaries around the delivery of care activities, with assistant nurses focused on clinical care and administrative tasks, and care aides carrying out personal care and some cleaning duties.

In our study, participants typically indicated that care tasks not part of their direct role duties were a lower priority. There was a common view that somebody else would attend to these low priority care tasks—they were someone else’s responsibility. This was particularly true for participants loading on Viewpoint 2 who were highly role-oriented. In a study of missed care in acute settings, Kalisch found that one of the seven themes related to reasons for missed care was task division based on role, termed “it’s not my job syndrome” [13]. This finding was supported by Kalisch’s later research assessing the relationships between unlicensed assistive personnel and registered nurses in hospital settings [51]. Registered nurses focused on the work only they were qualified to do, and were reluctant to engage with tasks they considered to be outside of their role.

#### Residents’ choices about their care

During data collection, participants advanced that it was important to them that residents were offered choices about their care, however other aspects of care needed to be prioritised for various reasons. This finding is supported by Simmons et al.’s work [52], in which staff members demonstrated a preference for affording residents choice, but could not always translate this preference to real-world contexts. Participants in Simmon et al.’s study discussed several barriers to the provision of choice, including residents’ dementia and staff members’ need to attend to residents’ physical health [52]. Other barriers to providing choice and autonomy to residents identified by previous research include a competing demand for safety, scheduled routines, and organisational policy and regulations [53–57]. These barriers align with the explanations participants in the current study gave for ranking choice-related cards as lower priorities. For example, although participants did not explicitly discuss the limitations of routines and organisational regulations on choice, they were very role-oriented, with their priorities influenced by their occupational position. As such, they may have tended to focus on regulated routines, role responsibilities and assigned care duties, forcing choice-related items to be ranked as lower priorities.

### Implications for practice and policy

The prioritisation of care based on role responsibilities, although often necessary (e.g., as dictated by government regulations, level of training, and qualifications), has practical implications regarding rationing of care and missed care. Our research shows that when care staff are highly focused on their assigned care duties, they place less priority on care tasks outside the scope of their role. Assuming other staff members will attend to a care activity (i.e., “it’s not my job syndrome”) means that lower priority tasks are susceptible to being missed. Furthermore, less concrete aspects of care, particularly those related to person-centredness such as offering residents choices about their care and conversations with residents, may be traded in favour of discrete tasks such as showering residents.

Participants’ perceptions of their job roles, and apparent division of labour, highlighted a systems-level issue regarding the training of residential aged care staff. In order to improve the safety and quality of care, staff training should incorporate a holistic approach to care provision. Participants loading on Viewpoint 1, particularly clinical staff members, expressed that residents were living in care facility because they needed assistance with their medical needs. However, there are a variety of reasons for transitioning into a residential aged care facility including the need for assistance with activities of daily living (e.g., toileting) or domestic tasks (e.g., cooking), reassurance of safety, and companionship/socialisation. Re-focusing training programs to promote care integration across services could better support staff to provide holistic care to residents and prevent care from being missed or neglected.

Internationally, there has been a push for a culture shift regarding the care provided to older populations, including those living in residential aged care facilities, from being institution-focused, to a more person-centred approach [58–62]. Person-centred care involves treating residents with dignity, engaging residents and their families in care planning and decision-making, designing care processes to meet the needs of residents, and respecting residents’ preferences and choices regarding their care [63]. In some countries, person-centred care in residential aged care facilities is mandated by government regulations, for example, Canada’s Residential Homes for Seniors Standards (resident-directed care) [64], England’s Health and Social Care Act 2008 Regulations (person-centred care) [65], New Zealand’s Health and Disability Service Standards (consumer rights) [66] and the United States of America’s Federal Code of Regulations (person-centred care) [67]. In Australia, residential aged care facilities must comply with the recently released Aged Care Quality Standards (July 2019) which include ‘consumer choice and dignity’ as the first of eight standards [68]. During the time of data collection, a previous set of quality standards were in effect. Relevant to residents’ choices about their care, standard 3.9 stated that “Each care recipient … participates in decisions about the services the care recipient receives, and is enabled to exercise choice and control over his or her lifestyle …” [69].

Our research demonstrated that despite policy requirements, and participants’ expressed desire to afford residents’ involvement in their care, residents’ choices were not prioritised by staff members. The view that residents’ choices are a lower priority than most other aspects of care has implications for quality of care and residents’ wellbeing. There is evidence that residents’ perceived autonomy and choice is negatively associated with depressive feelings [70], and positively associated with quality of life [70], life satisfaction [71], meal service satisfaction and nutritional status [72], and satisfaction with care preferences being met [56].

### Future research

Although assessment of the relationship between prioritisation and missed care was outside the scope of this study, our findings illustrated how assigning a lower priority to a care activity could lead to care being missed. Participants admitted that tasks that were not part of their assigned care duties were a lower priority and seen as someone else’s responsibility. Further investigations are warranted into the links between role-responsibilities, prioritisation of care, and missed care in residential aged care facilities, and the consequences these have for resident outcomes.

Another area for future research is the investigation of strategies used by care staff to avoid lower priority care from being missed or falling through the cracks. Our study indicated that one such strategy is the role Care Assistants hold as ‘knowledge brokers’. ‘Brokers’ are people within a network who connect other people or groups of people [27]. Specifically, ‘knowledge brokers’ transmit information and knowledge between people, facilitating the coordination of care [27].

### Strengths and limitations

Unlike other methods, for example, surveys, where participants independently assign each item a rating, Q methodology aims to produce a gestalt in which the interpretation of each card’s placement is considered in relation to every other card on the Q sort grid [32]. In this study, each participant’s resulting Q sort therefore represented an integrated and ‘whole’ view of care prioritisation. An additional strength of Q methodology is the integration of qualitative and quantitative data at the conceptualisation, data collection, analysis, and interpretation stages of research, situating the study as a fully integrated mixed design [73, 74].

Previous studies of prioritisation in residential aged care [27, 28], and related research on implicit rationing and missed care [7], have predominantly focused on the perspectives of nurses, physicians and carers. Our research acknowledged the multidisciplinary nature of residential aged care by involving other care staff providing direct care to residents: Managers, Pastoral Carers, and Activities and Lifestyle Officers. Other stakeholder groups such as allied health professionals, physicians, and agency staff members were not invited to participate in this study as they were not directly employed by the care organisation. Inclusion of these groups may have provided additional perspectives.

This study was conducted in five facilities across two Australian states, reducing the effects of facility-related context on study findings. Despite the variability in facility environments, all participating sites belonged to a single organisation. Seven participants declined the invitation to participate in the study, with time restrictions cited as the main reason for non-participation. This could have potentially introduced selection bias, however, with a participation rate of 81.6% (*n* = 31/38), it is unlikely to have had substantial impact on study findings.

## Conclusions

Our study identified four distinct viewpoints regarding care prioritisation in residential aged care facilities: Prioritisation of clinical care, Prioritisation of activities of daily living, Humanistic approach to the prioritisation of care, and Holistic approach to the prioritisation of care. Prioritisation of care was largely influenced by participants’ occupation and perceived role responsibilities. This finding has implications for missed care, as care activities viewed as falling outside the scope of participants’ assigned duties were consistently considered lower priorities. The division of care activities based on job role signifies that training programs should be adapted to incorporate more holistic and integrated approaches to care. Across the sample, participants consistently ranked residents’ choices regarding room environment, seating, clothes and meals as lower priorities. Our research suggests that despite government regulations pertaining to person-centred care, residents’ preferences regarding their care are often overlooked in favour of more task-specific aspects of care.

## Supplementary information

**Additional file 1.** Demographic questionnaire and semi-structured interview guide.

**Additional file 2.** Q cards, care categories and factor arrays.

**Additional file 3.** Visual representation of the factor array for Factor 1.

**Additional file 4.** Visual representation of the factor array for Factor 2.

**Additional file 5.** Visual representation of the factor array for Factor 3.

**Additional file 6.** Visual representation of the factor array for Factor 4.

## Data Availability

Data sharing is not applicable to this article as no datasets were generated or analysed during the current study.

## Abbreviations

MQRTP: Macquarie University Research Training Program
NSW: New South Wales
QLD: Queensland
