# Supplementary material for: Staff members’ prioritisation of care in residential aged care facilities: a Q methodology study
Source: BMC Health Serv Res. 2020 May 14;20:423. doi: 10.1186/s12913-020-05127-3 (PMC7222492; doi:10.1186/s12913-020-05127-3)
Supplement: Supplementary file 1 — Additional file 1. Demographic questionnaire and semi-structured interview guide. [file 12913_2020_5127_MOESM1_ESM.pdf]

Additional file 1: Demographic questionnaire and semi-structured interview guide

Demographic questionnaire

Age:
Gender:
Job title:
Length of time working at current organisation:

Semi-structured interview guide

1. At the beginning of each shift, how do you determine what your priorities are for that day?
2. How might your priorities change over the course of the day? What makes them change?
3. What things prevent you from attending to high-priority activities?
4. When you don't have enough time to complete all your required work in a shift, how do you manage your priorities?
5. What strategies do you use to make sure that care tasks that aren't carried out on time get completed?
6. How do you think your care priorities compare to the priorities of residents?
7. How do you think your care priorities compare to the priorities of residents' family members?
8. Is there anything else you want to talk about regarding the care you provide or care prioritisation?
