# Supplementary material for: Staff members’ prioritisation of care in residential aged care facilities: a Q methodology study
Source: BMC Health Serv Res. 2020 May 14;20:423. doi: 10.1186/s12913-020-05127-3 (PMC7222492; doi:10.1186/s12913-020-05127-3)
Supplement: Supplementary file 2 — Additional file 2. Q cards, care categories and factor arrays. [file 12913_2020_5127_MOESM2_ESM.pdf]

Additional file 2: Q cards, care categories and factor arrays

Card labels	Q card statements	Care category	Factor arrays—card rankings[a]			
			Factor 1	Factor 2	Factor 3	Factor 4
Assistance getting dressed	Assistance getting dressed when needed	Activities of daily living	-1*	+1	-3*	0
Assistance with meals	Assistance with meals when needed	Activities of daily living	+1	+4*	0	+2*
**Assistance with walking**	**Assistance with walking when needed**	**Activities of daily living**	**0**	**+1**	**0**	**+1**
Attitudes towards family	The facility/home is welcoming to family members	Psychosocial care	0	-2	+1*	-4*
Bathing and showering	Assistance with bathing/showering when needed	Activities of daily living	+2	+3*	-1*	+1
**Bowel care**	**Bowel care is provided when needed**	**Activities of daily living**	**+1**	**0**	**0**	**+1**
Call bell	Residents' call bells are responded to in a timely manner	Clinical care	+2	0	+1	0
**Choice about room environment**	**Residents have choice about what is in their room**	**Independence and choice**	**-4**	**-4**	**-2**	**-3**

Clothing changed	Residents' clothes are changed when needed	Activities of daily living	-3	0	-2	-2
Clothing choice	Residents have choice about the clothes they wear	Independence and choice	-2	-1*	-4	-3
Conversations	Time is taken to chat with residents	Psychosocial care	-2	-2	+2	+1
Emotional support	Emotional support is provided to residents	Psychosocial care	-1**	+1*	+4*	+3*
Family information	Family members are informed about residents' medical care	Clinical care	+1	-3**	+1	+1
Independence	Residents have independence	Independence and choice	-1	-1	+2	+2
Meal choice	Residents have choice about their meals	Independence and choice	-2	0*	-2	-2
Medical condition management	Residents' medical conditions are managed	Clinical care	+3	-3	+3	+4
Medication management	Correct medication at the right time	Clinical care	+4	+2**	+1	+3
Mobility	Residents are supported to keep active and mobile	Clinical care	-1	-1	0	0

Monitoring/ Safety	Residents are kept safe from injury or medical harm	Clinical care	+4	+2	+2	+4
Nail care	Nail care is provided when needed	Activities of daily living	-3	-1	-3	-4
Nutrition	Residents' meals are nutritious	Clinical care	+1	+2	-1	-1
Oral care	Regular dental and oral care is provided	Activities of daily living	+0	+4**	-1	-1
Personal grooming	Assistance with personal grooming when needed	Activities of daily living	-1	+2**	-1	-1
Privacy	Residents' privacy is respected	Respect	+2	+3	+3	-1**
**Repositioning**	**Assistance with repositioning when needed**	**Activities of daily living**	**0**	**0**	**-1**	**0**
**Resident decision-making**	**Residents are involved in making decisions about their care**	**Clinical care**	**+1**	**0**	**+2**	**0**
Resident information	Residents are informed about their medical care	Clinical care	+3**	-4**	0	-1
**Respect**	**Residents are treated with respect**	**Respect**	**+2**	**+1**	**+4**	**+3**
Seating choice	Residents can choose where to sit	Independence and choice	-4	-2	-4	-3

	during group activities					
Skin care	Skin care is provided when needed	Activities of daily living	0	+1	-2	-2
Social activities	Residents are offered a variety of social and leisure activities	Psychosocial care	-2	-2	-3	0**
Spiritual activities	Opportunities for residents to engage in spiritual activities	Psychosocial care	-3	-3	+3**	-2
Staff knowledge	Staff are knowledgeable about residents' medical care	Clinical care	+3**	-1*	+1	+2
Toileting	Assistance with toileting needs	Activities of daily living	0	+3	0	+2

[a]This column presents card rankings on the Q sort grid for each factor array. For example, 'Assistance with meals' was ranked at +4 for Factor/Viewpoint 2, signifying a high priority. Comparatively, this card was ranked at 0 for Factor/Viewpoint 3, suggesting that this is neither a high nor low priority.

* Distinguishing statement at $p<0.05$

** Distinguishing statement at $p<0.01$

Consensus statements at $p>0.05$ are bolded
