# Supplementary figures and images for: Staff members’ prioritisation of care in residential aged care facilities: a Q methodology study
Source: BMC Health Serv Res. 2020 May 14;20:423. doi: 10.1186/s12913-020-05127-3 (PMC7222492; doi:10.1186/s12913-020-05127-3)

### Additional file 3: Visual representation of the factor array for Factor 1

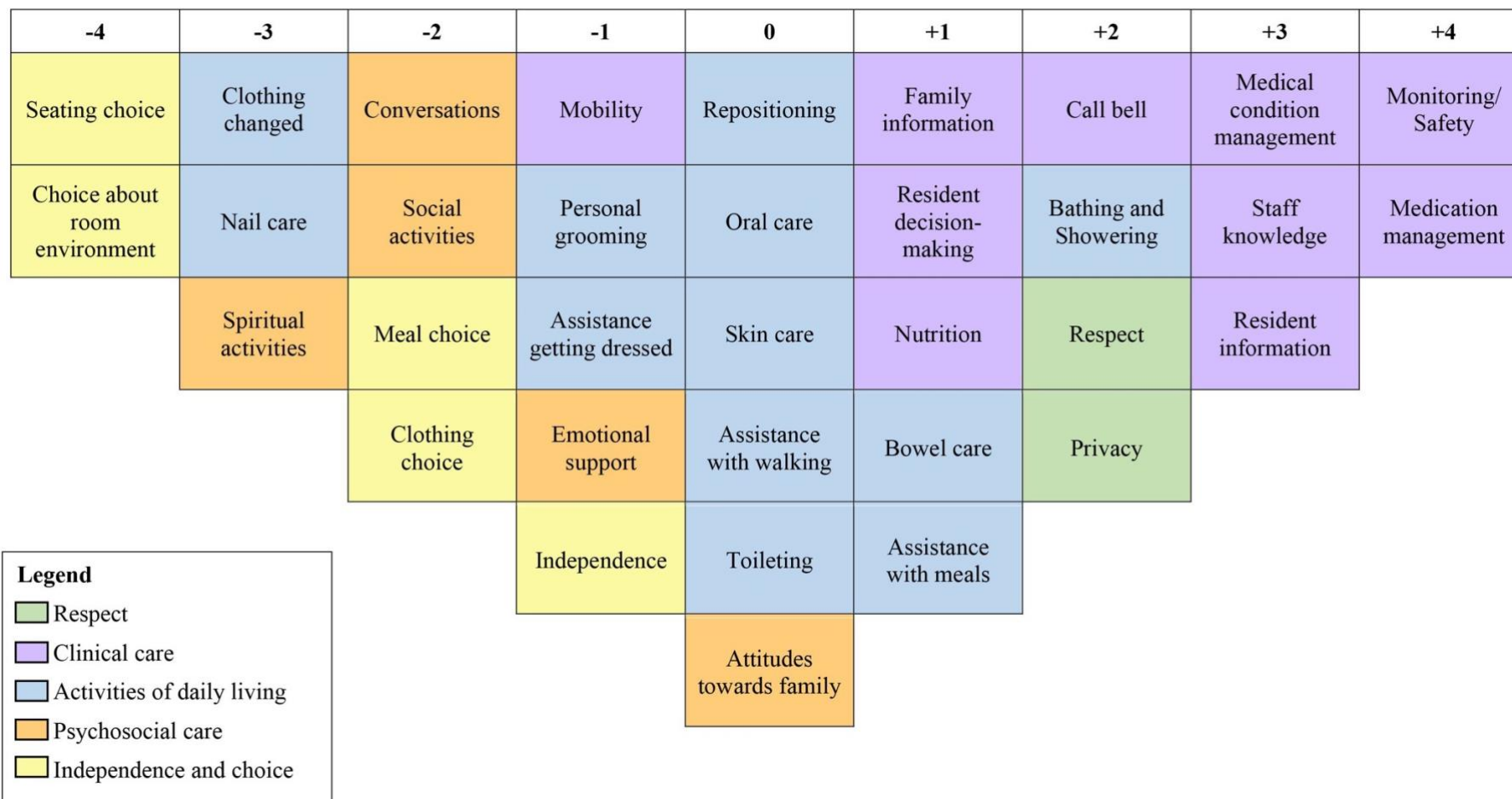

Supplement: Supplementary file 3 — Additional file 3. Visual representation of the factor array for Factor 1. [file 12913_2020_5127_MOESM3_ESM.pdf]

**Additional file 4: Visual representation of the factor array for Factor 2**

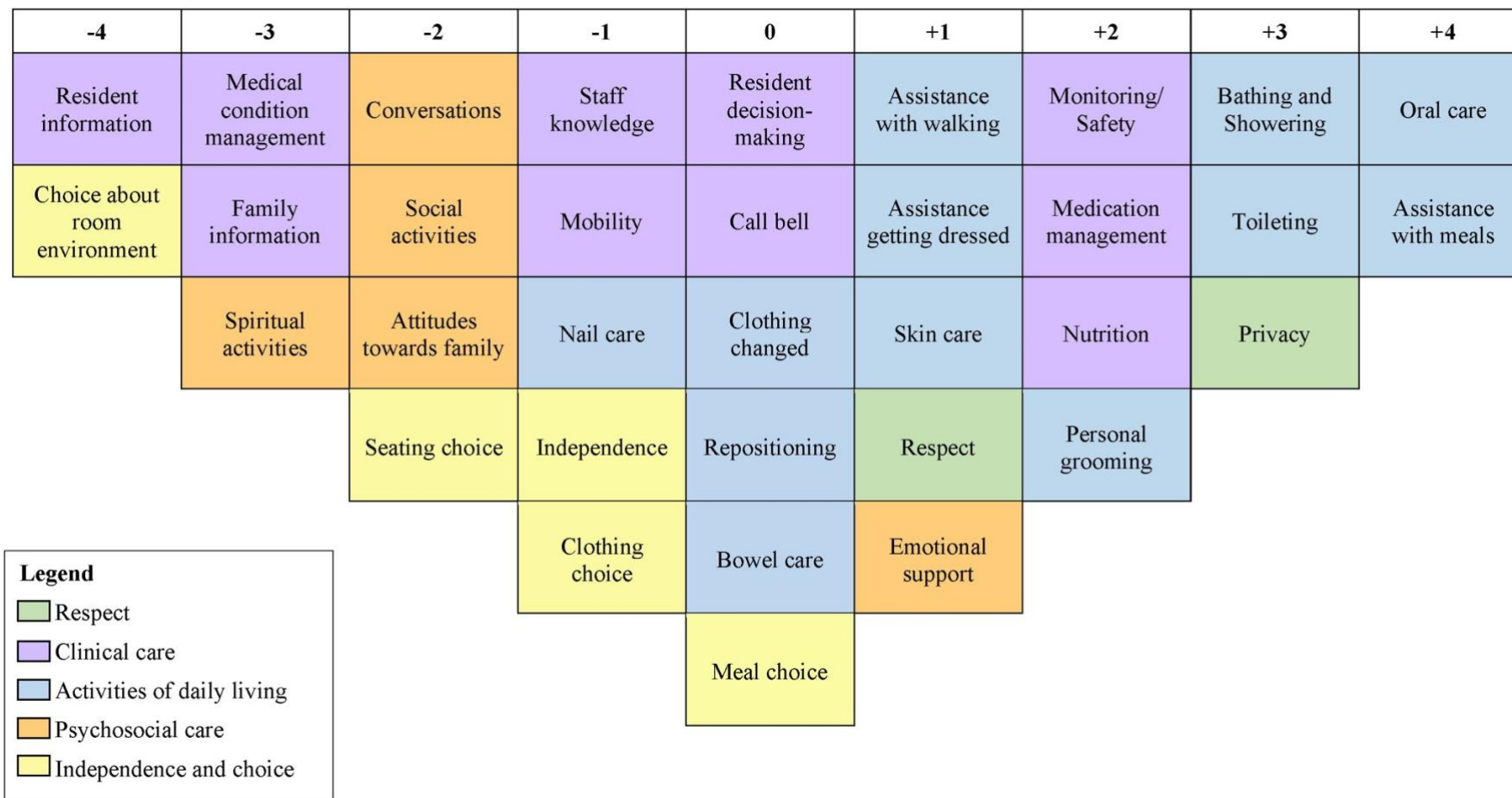

Supplement: Supplementary file 4 — Additional file 4. Visual representation of the factor array for Factor 2. [file 12913_2020_5127_MOESM4_ESM.pdf]

**Additional file 5: Visual representation of the factor array for Factor 3**

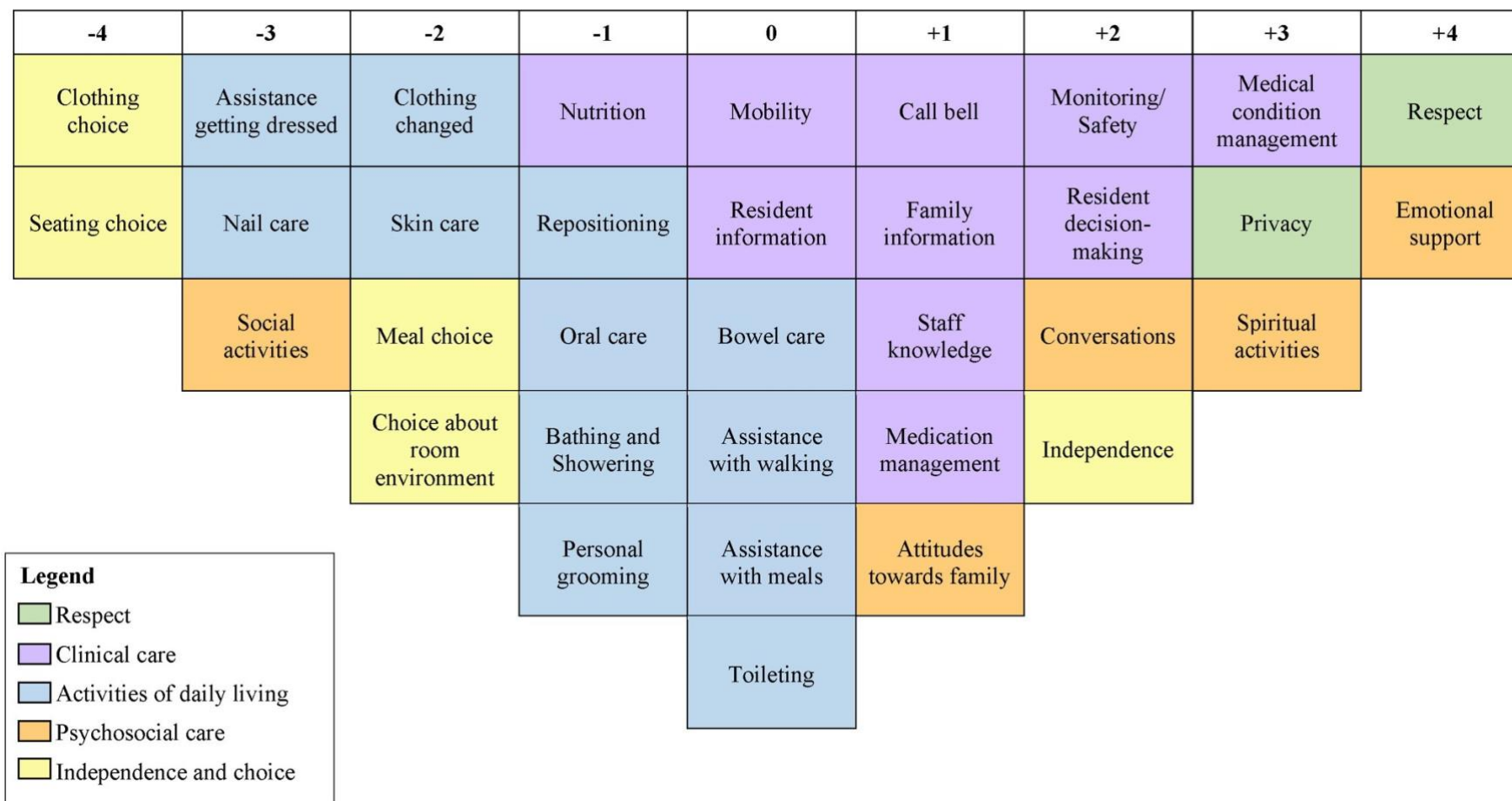

Supplement: Supplementary file 5 — Additional file 5. Visual representation of the factor array for Factor 3. [file 12913_2020_5127_MOESM5_ESM.pdf]

**Additional file 6: Visual representation of the factor array for Factor 4.**

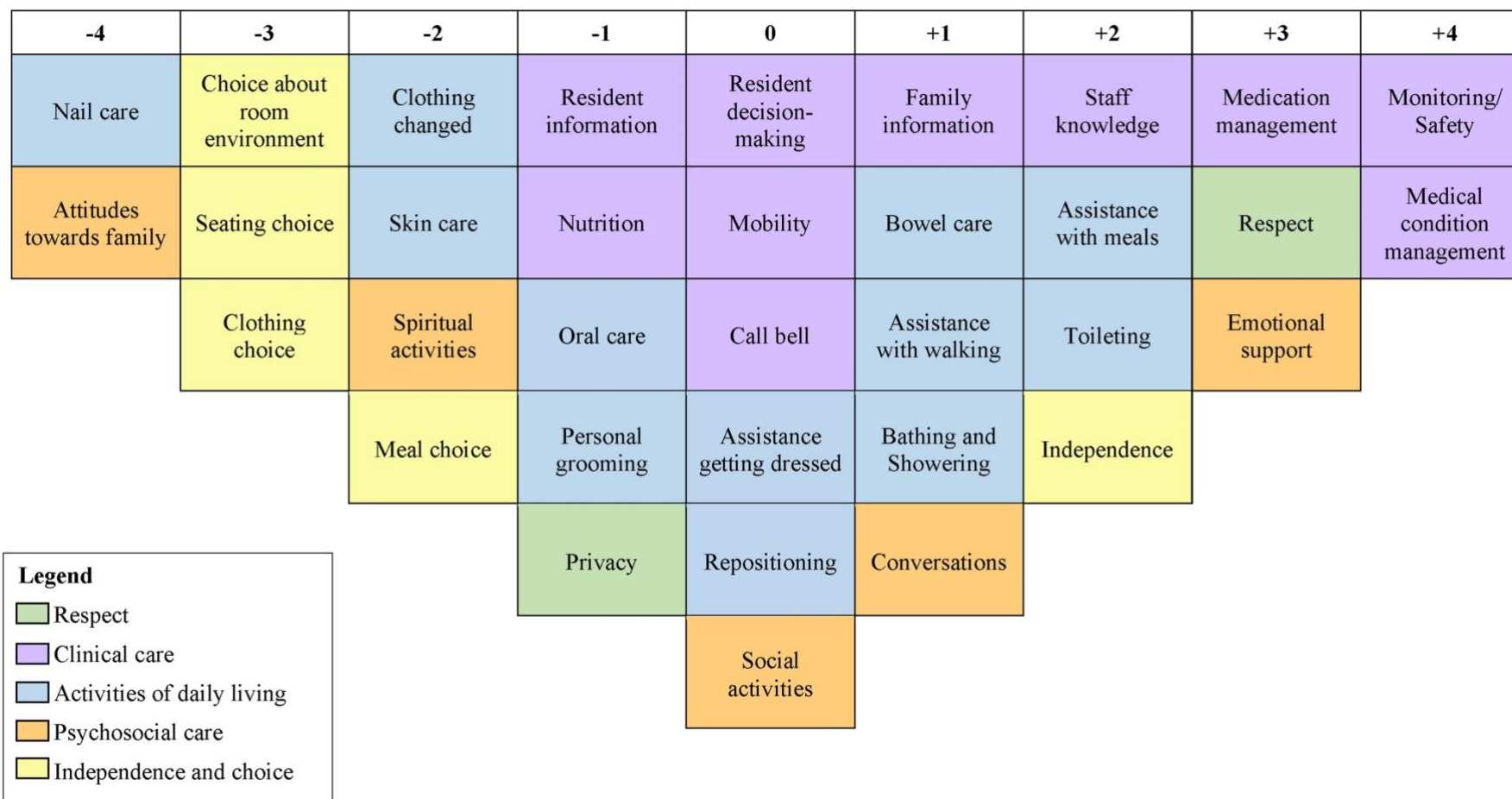

Supplement: Supplementary file 6 — Additional file 6. Visual representation of the factor array for Factor 4. [file 12913_2020_5127_MOESM6_ESM.pdf]
